# Hemi-central retinal artery occlusion following methanol toxicity

**DOI:** 10.3205/oc000207

**Published:** 2022-11-16

**Authors:** Mohammad Zarei, Sima Sheikhghomi, Masoud Aghsaei Fard

**Affiliations:** 1 Farabi Eye Hospital, Tehran University of Medical Sciences, Tehran, Iran; 2 Shahid Madani Hospital, Alborz University of Medical Sciences, Karaj, Iran

**Keywords:** hemi-central retinal artery occlusion, methanol toxicity

## Abstract

**Objective:**

To describe a patient with hemi-retinal artery occlusion following methanol toxicity.

**Methods:**

Observational case report.

**Results:**

We report a case presented with an acute altitudinal visual field loss in the right eye following consumption of illicit alcoholic drink. In fundus photography, a well demarcated superior hemi-retinal whitening with foveal sparing was noted. Careful inspection of the optic nerve head in the right eye revealed that there was no main trunk of the central retinal artery anterior to the lamina cribrosa. Two separately emerging superior and inferior arterial trunks were noted. In fundus fluorescein angiography, earlier dye filling in the territory of the superior arterial trunk compared to the inferior arterial trunk was evident.

**Conclusion:**

Hemi-central retinal occlusion may happen as an ocular consequence of methanol toxicity in patients with a proximal bifurcation of the central retinal artery.

## Introduction

Poisoning with illicit alcoholic drinks is a common problem in some developing countries, where recreational alcoholic consumption is forbidden due to legal constraints. In September and October of 2018, an epidemic of methanol toxicity related to homemade alcoholic drinks happened in some cities of Iran [1]. Following absorption, methanol is metabolized into formaldehyde and then formic acid, which causes metabolic acidosis [2]. Ophthalmologically, methanol poisoning leads to blurred vision due to a toxic optic neuropathy which is usually associated with optic disc edema and/or retinal swelling [3], [4]. Herein, we report for the first time a case of superior hemi-retinal arterial occlusion following poisoning with illicit homemade alcoholic drink during the aforementioned epidemic.

## Case description

A 20-year-old man presented with a complaint of sudden, painless inferior visual field loss in his right eye that he had noticed since a few days ago. He denied any remarkable previous ocular or systemic diseases but had consumed a homemade alcoholic drink one week earlier with two of his friends. Subsequently, all of them experienced severe nausea and vomiting. Forty-eight hours after consumption, one of them became comatose and was transferred to a referral poisoning center, and was treated for methanol toxicity and metabolic acidosis with mechanical ventilation and hemodialysis. After regaining consciousness, he noticed blurred vision and with the diagnosis of methanol induced optic neuropathy was treated with intravenous methylprednisolone and erythropoietin [5], [6], [7]. Six days later, on ophthalmic examination in our clinic, a visual acuity of 20/200 in each eye was documented. 

**Figure 1:**
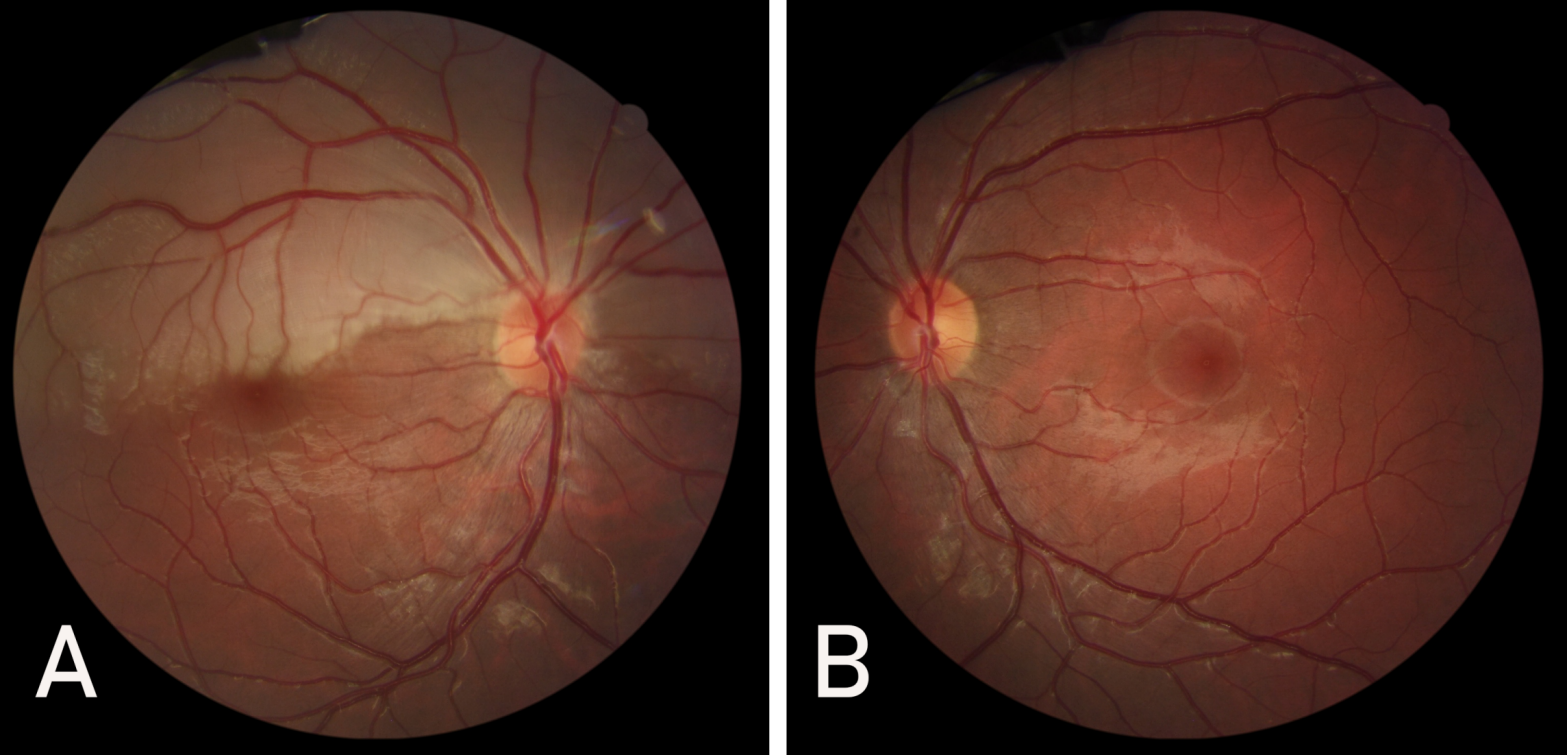
A: A well demarcated superior hemiretinal whitening and foveal sparing in the right eye. B: Normal fundus appearance of the left eye.

**Figure 2:**
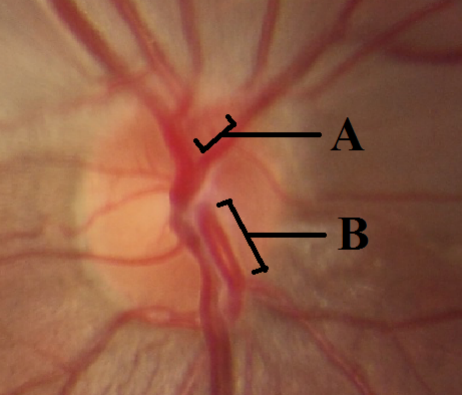
Fundus photography of the right optic nerve head showing separated emergence of the superior and inferior hemi-trunks of the central retinal artery. A: superior hemi-trunk of the central retinal artery. B: inferior hemi-trunk of the central retinal artery.

**Figure 3:**
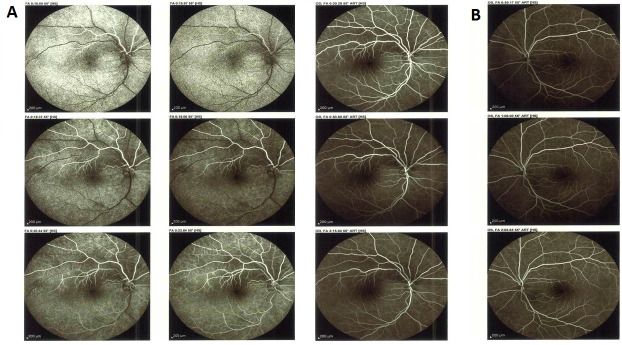
A: Fundus fluorescein angiography of the right eye displaying the earlier dye filling of the superior arterial hemi-trunk and its main branches in the choroidal phase. Dye appearance in inferior retinal arterial vasculature occurred a few seconds later in the arterial phase. B: Fluorescein filling of the left eye. No filling defect was detected in eyes.

**Figure 4:**
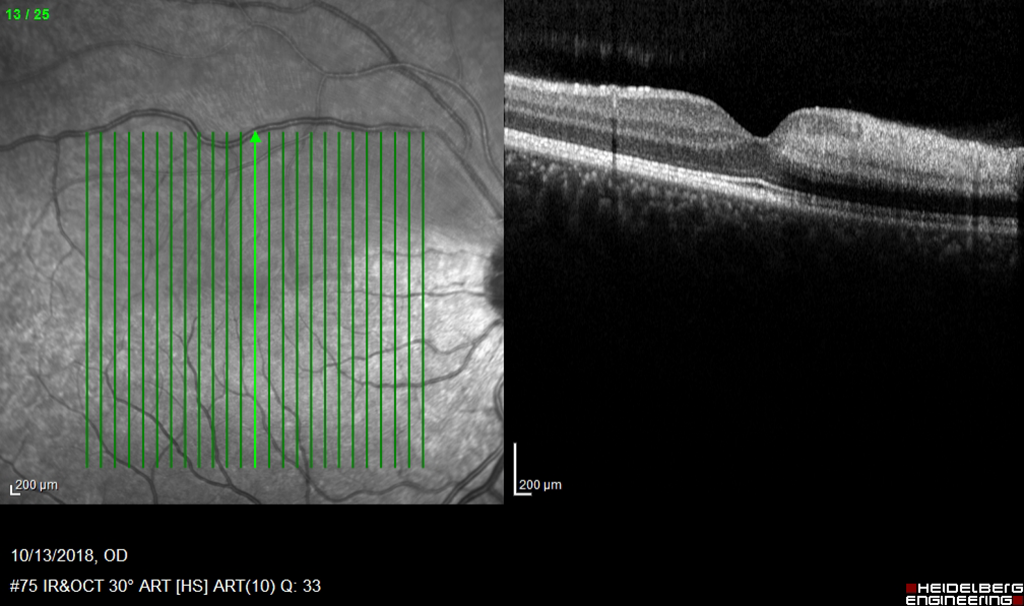
Vertical macular optical coherence tomography of the right eye; thickening and hyperreflectivity of the inner superior macula is a consequence of ischemia caused by superior hemi-central retinal artery occlusion.

In contrast to his friend, our patient reported no loss of consciousness or visual decline except of an acute visual field loss in the right eye. However, with the assumption that the problem may resolve on its own, he did not seek for medical care immediately. On presentation, visual acuity was 20/20 in both eyes, but a relative afferent pupillary defect was present in the right eye. In fundus photography of the right eye, a well demarcated superior hemi-retinal whitening involving the superior half of the macula with foveal sparing was noted while fundus appearance was completely normal in the left eye (Figure 1 (Fig. 1)). On a more precise inspection of the optic nerve head of the right eye, two separately emerging superior and inferior arterial trunks were noted and no main trunk of the central retinal artery was detected anterior to lamina cribrosa (Figure 2 (Fig. 2)). Fundus fluorescein angiography (FFA) of the right eye revealed an early arterial filling in the superior arterial trunk and its main branches in the choroidal phase. Dye appearance in inferior retinal arterial vasculature occurred a few seconds later in the arterial phase. Fluorescein filling of the superior and inferior arterial trunks of the left eye was simultaneous. No filling defects were detected in either eye (Figure 3 (Fig. 3)). Macular optical coherence tomography (OCT) showed increased reflectivity of the inner layers in the superior macula (Figure 4 (Fig. 4)). A corresponding visual field defect was documented in automated perimetry. 

On repeated questioning, the patient denied any history suggestive of thrombophilia or migraine. Count of blood cells, renal and liver function tests and coagulation profile were unremarkable. In an attempt to find the origin of the presumed thromboembolism, a cardiovascular evaluation, including cardiac exam, echocardiography, and Doppler sonography of the cervical vessels were performed and no abnormality was found. Three weeks later, no improvement in the visual field was observed.

## Discussion

As the first branch of the ophthalmic artery, the central retinal artery (CRA) enters the optic nerve tissue about the 8–15 mm behind the lamina cribrosa inferolaterally or inferomedially. Thereafter, CRA continues its course towards the globe and after passing through the lamina cribrosa, divides into superior and inferior trunks while histologically loses its elastic lamina [8], [9]. Occasionally, this division occurs behind the lamina cribrosa and the two trunks emerge on the optic nerve head as separate arteries. Hayreh et al. have explained this anatomy and the fact that behind the lamina cribrosa a superior intraneural artery which lacks elastic lamina may arise from the central retinal artery which supplies the superior half of the retina [10]. As a result, it can be understood why most of the hemi-central retinal artery occlusions (hemi-CRAOs) occur in the superior retina. 

Rishi et al. reported 4 patients with hemi-CRAO whose superior and inferior retinal arteries appeared separated at the optic disk without any detectable main central trunk. They further investigated their patients with color Doppler imaging of the optic nerve head and localized the arterial bifurcation just behind the lamina cribrosa. They also reported that the fundus fluorescein angiography of these patients revealed early filling of the reperfused arterial trunk [11]. Moreover, Schmidt et al. also reported this early dye filling of the involved arterial branch in FFA of their patient with a drug induced hemi-CRAO. They stated that this paradoxical finding would be a sequel of the vascular wall ischemia, which leads to luminal dilation, and consequently, according to Hagen-Poiseuille’s law, the blood flow is increased in these dilated vessels [12]. It can be inferred that although it is more expectable to encounter an arterial filling defect in the acute phase of the hemi-CRAO, the reperfused arterial trunk may show earlier filling as soon as it reopens. 

Regarding our patient, although early filling of the superior half of the retina could be misleading toward a cilioretinal artery occlusion, prescence of the two separate emerging arterial trunks within the optic cup, lack of characteristic appearance of ciliaoretinal artery, and the absence of contribution of this branch to blood supply of the optic disk exclude the diagnosis of cilioretinal artery occlusion [13].

Cases of hemi-central retinal artery occlusion with various systemic underlying conditions have been reported: congenital cyanotic heart disease, moyamoya syndrome, hypercoagulability conditions such as polycythemia vera or mutation of factor V Leiden, artificial mitral valve, giant cell arteritis, iatrogenic causes (such as endovascular embolization procedures), and consumption of some drugs (including Sildenafil, Anastrozole, oral contraceptive pills) [14], [15], [16], [17], [18]. According to several experimental studies, internal elastic lamina protects against blood pulse pressure. However, the central retinal artery loses this layer as it bifurcates and lamina cribrosa becomes crucial in the autoregulation of these vessels. Therefore, early bifurcation of the central retinal artery proximal to lamina cribrosa exposes these vulnerable vessels with imperfect autoregulation to physiological and pathological triggers [19], [20]. 

This is the first case report of hemi-CRAO following methanol toxicity in a healthy young patient without any other detected predisposing condition. The rare occurrence of unilateral superior hemiretinal arterial occlusion in a young healthy man in few days after consumption of methanol-containing alcoholic drink should be considered more than a coincidence. In methanol poisoning, metabolic acidosis from the accumulation of formic acid results in neural and vascular injury. It is often asserted that poisoning disrupts mitochondrial function especially in the optic nerve and results in optic nerve swelling and loss of vision. 

In this case, it is assumed that methanol-induced swelling affected the superior arterial hemi-trunk preferentially due to its aforementioned anatomic and histologic variation. 

Management of the hemi-CRAO consists primarily of controlling the underlying disease to prevent future vascular accidents. Generally, no effective and proven treatment exists to treat the permanent loss of the visual field or acuity [21], [22], [23]. 

## Conclusion

We suggest that hemi-CRAO may happen as an ocular consequence of methanol toxicity in patients with a proximal bifurcation of the central retinal artery.

## Notes

### Ethics statement

This case report was conducted in accordance with the Declaration of Helsinki. The authors obtained written informed consent from the patient for publishing the information and images.

### Acknowledgment

The authors would like to thank Dr. Nasim Zamani for her critical contribution to this report.

### Competing interests

The authors declare that they have no competing interests.
